# Trade liberalization, social policies and health: an empirical case study

**DOI:** 10.1186/s12992-015-0126-8

**Published:** 2015-10-12

**Authors:** Courtney McNamara

**Affiliations:** Department of Sociology and Political Science, Norwegian University of Science and Technology, Building 9 level 5, Dragvoll, 7491 Trondheim, Norway

**Keywords:** Trade, Social policy, Labor markets, Social determinants

## Abstract

**Background:**

This study investigates the health impacts of a major liberalization episode in the textile and clothing (T&C) sector. This episode triggered substantial shifts in employment across a wide range of countries. It is the first study to empirically link trade liberalization to health via changes in employment and offers some of the first empirical insights on how trade liberalization interacts with social policies to influence health.

**Methods:**

Data from 32 T&C reliant countries were analysed in reference to the pre- and post-liberalization periods of 2000–2004 and 2005–2009. Fuzzy-set qualitative comparative analysis (fsQCA) was used to examine the association between countries’ a) level of development b) labour market and welfare state protections c) T&C employment changes and d) changes in adult female and infant mortality rates. Process tracing was used to further investigate these associations through twelve in-depth country studies.

**Results:**

Results from the fsQCA relate changes in employment after the phase-out to both changing adult female and infant mortality rates. Findings from the in-depth country studies suggest that the worsening of adult female mortality rates is related to workers’ lack of social protection, both in the context of T&C employment growth and loss.

**Conclusions:**

Overall, it is found that social protection is often inaccessible to the type of workers who may be the most vulnerable to processes of liberalization and that many workers are particularly vulnerable due to the structure of social protection policies. Social policies are therefore found to both moderate pathways to health and influence the type of health-related pathways resulting from trade liberalizing policies.

**Electronic supplementary material:**

The online version of this article (doi:10.1186/s12992-015-0126-8) contains supplementary material, which is available to authorized users.

## Background

The health impacts of trade liberalization have begun to receive more attention in public health scholarship. However, research thus far has focused largely on the direct impacts of biomedical or lifestyle factors. Trade liberalization’s impacts on the social determinants of health (SDH) have by comparison received little consideration [1].

SDH constitute the social conditions that shape people’s ability to lead healthy lives [2]. They include factors like income, education and employment. Encompassing the reduction of tariffs, quotas and other barriers to trade, trade liberalization can directly affect the distribution of these and other SDH [1]. However, social policies also contribute to SDH, directly through transfers and services, like unemployment insurance and pensions, and indirectly through policies that affect people’s labour market opportunities [3]. Therefore, the extent to which trade liberalization impacts health will depend not just on the characteristics of trade policies but also on the characteristics of states’ social policies.

The purpose of this study was to investigate the health impacts of a major trade liberalization episode in the textile and clothing (T&C) sector: the phase-out of the Multi-Fibre Arrangement (MFA) in 2005. This phase-out triggered substantial shifts in employment across a wide range countries and thus represents a valuable opportunity for exploring the health impacts of liberalization through an important SDH pathway. Since systems of social protection in impacted countries are highly diverse, analysing how health outcomes changed after the MFA phase-out can also help to develop our understanding of how trade liberalization and social policies interact to influence health.

This study is situated at the cross-roads of two areas of public health research. The first deals with the impact of trade processes on health, the second is concerned with how social policies influence cross-national variations in health. The intersection of these two areas has remained largely unexamined. The contribution of this research is thus three-fold. First, it contributes to our understanding of causal pathways surrounding trade and an important SDH: employment. Second, it provides the first empirical examination of the relationship between trade liberalization, social policies and health. Third, it employs an original and innovative method: fuzzy-set qualitative comparative analysis (fsQCA) combined with in-depth country case-studies. In doing so, this work not only demonstrates the merits of an under-utilized method in public health, but also offers some of the first empirical evidence of how social policies can both moderate and influence the type of health-related pathways resulting from trade liberalizing processes.

### Trade liberalization, labour markets and health

Two bodies of literature provide the theoretical background of this work. The first ties trade liberalization to health through labour market conditions such as wages, working conditions, job loss and economic insecurity (e.g., [4–8]). However, these connections are often made at the theoretical level. While the pathways between many of these conditions and health are well-grounded in empirical work [9], there is little empirical evidence which directly links trade liberalization to labour markets and in turn, health.

The second body of literature relevant to this work demonstrates that health outcomes vary significantly across different welfare state arrangements [10], where ‘*welfare state*’ is a term that describes the characteristics of a state’s social policy [11]. Generally speaking, this body of work finds evidence for the health importance of social protection policies [10]. However, these studies often assume that countries have complete control over processes that impact broad SDH, such as levels of employment [12]. The role of trade policy in limiting countries’ policy space surrounding these determinants is thus overlooked [13].

**Fig. 1:**
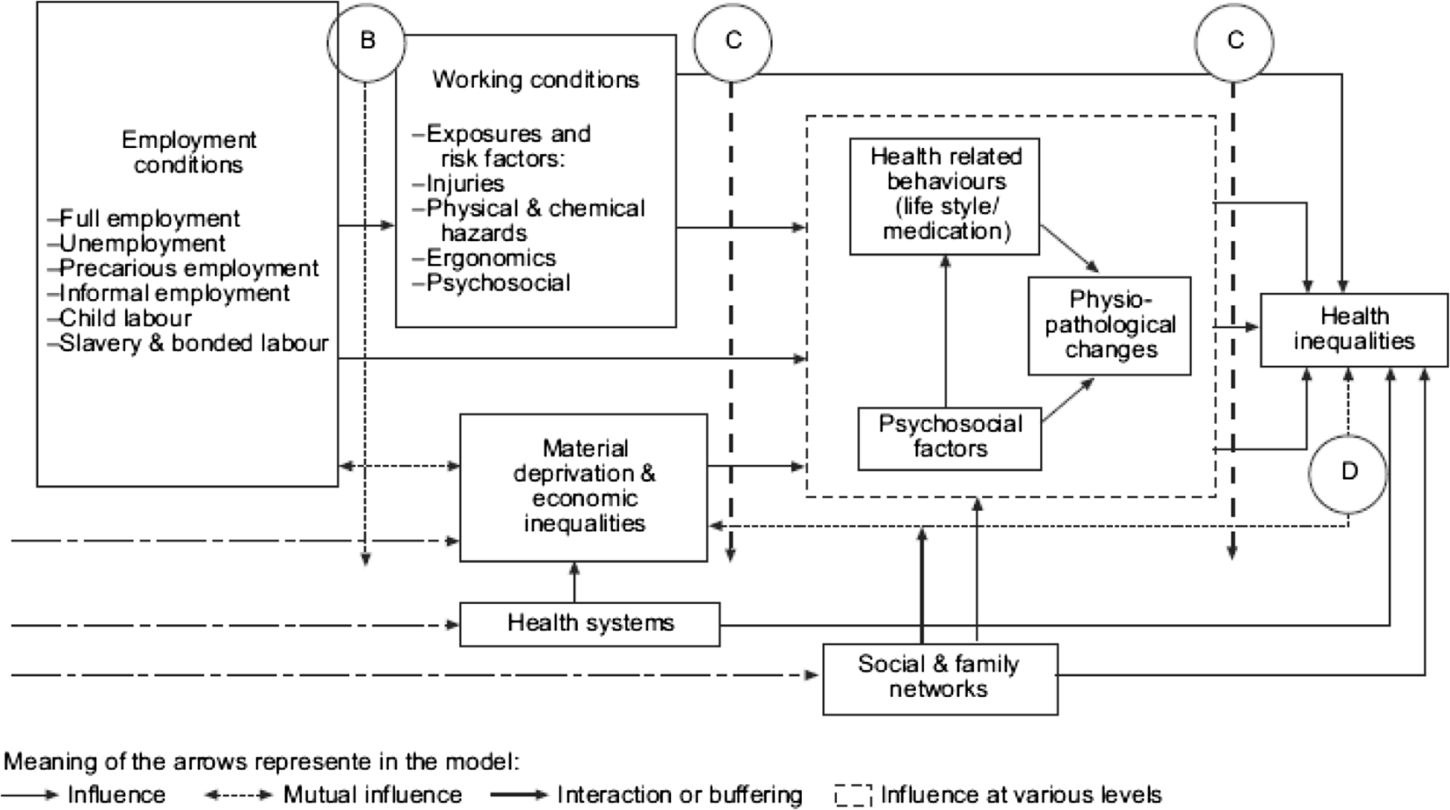
Micro-level framework

**Fig. 2:**
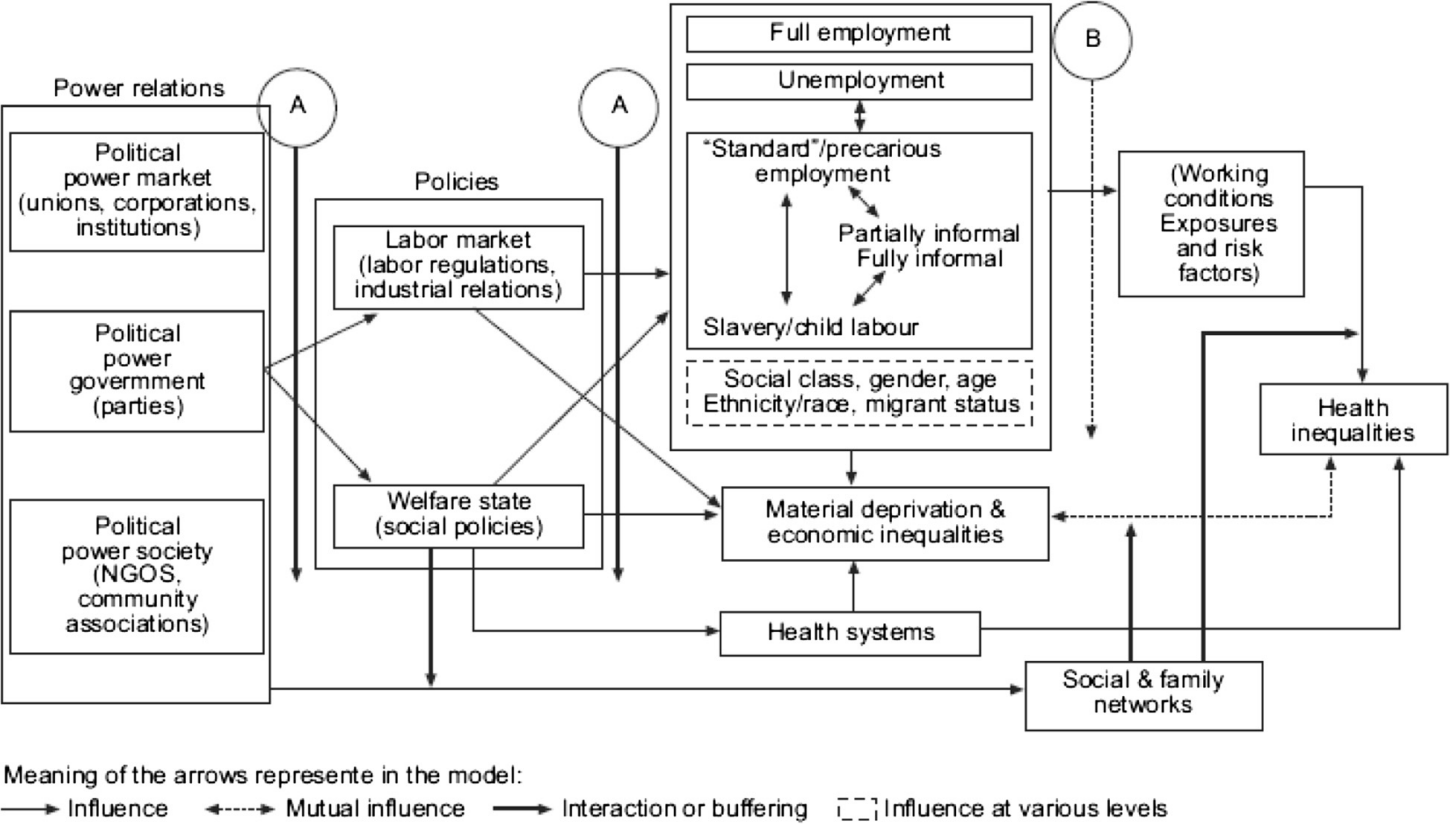
Macro-level framework

A framework by the Employment and Working Conditions Knowledge Network (EMCONET) of the World Health Organization’s Commission on the Social Determinants of Health, is unique in bringing these two bodies of literature together [14]. Figures 1 and 2 illustrate this framework.

Within the micro-level, different categories of risk exposure are mediated by social mechanisms and influenced by various types of employment and working conditions. At the macro-level, the framework focuses on the role of protective social policies, specifically, labour market and welfare state policies, in shaping these more micro-level factors. While trade liberalization is not explicitly depicted within the framework, the authors identify liberalization as one of the main drivers of conditions within this macro-level context.

### The textile and clothing sector

Prior to 2005, the T&C sector was based on a system of quotas. This system determined how many T&C items countries could export to the large importing markets of the E.U., U.S. and Canada. Quotas created incentives for companies in countries meeting their own export limits to set up production facilities in other, less constrained, countries [15]. Because the allocation of quotas was significantly more lenient towards countries with little or no T&C industry, high-quota countries saw a significant expansion of their industry [16].

Restrictions on T&C goods began in the 1950s and were formalized through a series of agreements culminating in the Multi-Fibre Arrangement in 1974. The MFA aimed to gradually open the T&C sector [17] however, subsequent negotiating rounds resulted in increasingly restrictive quotas [18]. In 1994, the Agreement on Textiles and Clothing established that countries wishing to retain quotas would have to commit to a ten-year phase-out period. This phase-out was meant to take place gradually, with the last quotas lifted on January 1, 2005. However, using ‘safegaurd’ measures, countries were able to maintain the majority of their quotas until the final phase-out period. The US maintained about 89 % of its quotas until this date; the EU, 70 % and Canada, 79 % [19]. Liberalization of the sector was therefore both abrupt and rapid.

Following the final phase-out, countries saw significant changes in their T&C employment [15]. Between 2004 and 2008, for example, India and Bangladesh saw their T&C employment grow by 21 and 40 %, respectively. Mexico, by contrast, saw its employment shrink by 35 % and Romania by nearly 40 % ([15]; author’s calculations using [20]).

### Research question

This study uses the EMCONET framework as a heuristic tool for thinking about how the MFA phase-out might have impacted health. Because the framework indicates a complex set of potentially relevant pathways, it is difficult to predict precisely how health may have been influenced by T&C employment changes. For example, employment growth after the phase-out might have facilitated better health via improved wages and material conditions. However, because poor working conditions characterize much of the work in the sector, there are also significant ways in which health may have been negatively impacted. In terms of employment loss, decreased exposure to poor working conditions may have positively impacted health while loss of income may have facilitated health deterioration through worsening material circumstances.

In total, the EMCONET framework suggests that employment changes after the MFA phase-out were likely to have combined with other causal conditions to influence health. Importantly, it also indicates that these other conditions are ultimately shaped by the protectiveness of countries’ welfare state and labour market policies. Therefore, the central research question this article is concerned with is how T&C employment changes after the MFA phase-out combined with countries’ labour market and welfare state policies to influence changes in health.

## Methods

To answer this study’s research question, the method of fuzzy-set qualitative comparative analysis (fsQCA) was combined with in-depth country case-studies using process tracing methods.

FsQCA is a subset of qualitative comparative analysis (QCA) which is a configurational approach focused on whether specific combinations of causal conditions (i.e., configurations) are associated with an outcome [21]. Configurational methods recognize that 1) outcomes are produced via a combination of conditions, 2) the same outcome may be produced by different combinations of conditions, and 3) the context within which conditions combine can influence the impact made on an outcome [22]. As demonstrated by recent studies (e.g., [23, 24]), configurational analyses can be particularly attractive for public health researchers when health outcomes are seen to be the result of a complex interplay of causal and contextual conditions. Such an approach is especially appropriate for this study since it is expected that employment changes after the MFA phase-out will combine with countries’ labour market and welfare state policies in different ways to influence health.

In contrast to regression models, which use correlational analyses to identify average effects, QCA uses set-theory to make logical statements about causal conditions—both alone or in combination--that are necessary and/or sufficient for an outcome [21]. A necessary relation exists if an outcome is a subset of a causal condition. Thus a condition is considered necessary if all (or virtually all) instances of the outcome show the condition. A sufficient relation exists if a causal condition, or combination of conditions, is a subset of an outcome. A condition (or combination thereof) is thus considered sufficient if an outcome always (or virtually always) occurs when a causal condition is present (although other conditions may also produce the outcome).

QCA techniques can be used for different purposes such as the testing of specific hypotheses, data exploration or for theoretical development [25]. This study uses QCA primarily for theoretical development since existing theory surrounding trade liberalization, labour markets and health remains broad and imprecise. Towards this end, a main advantage of QCA is that it can offer valuable insights into the causal processes shaping a relationship between causal conditions and an outcome. This relates in part to the dual nature of the approach which can be described as having both quantitative and qualitative features. Like mainstream statistical analyses, for example, cases differ quantitatively across causal conditions. In contrast to these methods however, QCA specifies thresholds at which these quantitative differences denote a causally important, qualitative difference. For example, whereas regression techniques assume that a causal condition will have an incremental impact on an outcome across all levels of variation in that condition, QCA specifies a point at which the condition begins to have a causal influence. QCA then sets to examine how qualitative differences across cases are associated with an outcome. This qualitative focus on the causal conditions can shed light on key elements of the mechanisms and processes behind necessary and/or sufficient relations.

The qualitative features of QCA also mean it can be used to identify specific types of cases for detailed within-case analyses which in turn can offer insight on the results of the QCA and its surrounding theory [26]. This can further aid in theory development and help overcome one of the main weaknesses of QCA, i.e., the fact that like regression techniques, it identifies associations not causation [26]. It is for these reasons that this study combined fsQCA with in-depth country case-studies using theory-building process tracing methods. Theory-building process tracing is an approach which can be used both to further explore the details of cases and to construct potential causal mechanisms in scenarios where we see associations but theory is unable to offer precise ideas about the causal processes behind them [27].

### FSQCA analysis

FsQCA is carried out in three steps. First, outcome indicators, cases and causal conditions are identified. Included cases are then assigned membership scores for each of the outcomes and causal conditions. In a conventional QCA, cases are either members of the set created by the indicator (with a membership score of 1) or not (with a membership score of 0). In fsQCA by contrast, cases can have partial membership anywhere in the range of 0-1 [25]. It was decided to use fsQCA instead of conventional (i.e., ‘crisp-set’) QCA since cases in this study are better characterized through their degree of membership in the causal conditions under consideration.

In the second stage of fsQCA, examinations of necessity and sufficiency are undertaken. Here a truth table is constructed which outlines the empirical instances of configurations, as well as their relationship to the outcome indicators. With fsQCA there are 2^k^ possible configurations, where k represents the number of causal conditions.

Because it is rare for cases to conform precisely to either a necessary or sufficient relation, the concepts of consistency and coverage are used to measure how well this is achieved [28, 29]. Consistency measures the degree to which a necessary/sufficient relation is met. In terms of necessity, consistency measures the degree to which an outcome is a subset of a causal condition. If all instances of the outcome display the causal condition, consistency will be high. In terms of sufficiency, consistency measures the degree to which a causal condition (or combination of) is a subset of an outcome. If all instances of the condition display the outcome, consistency will be high. Scores are calculated by the fsQCA software and range from 0 (no consistency) to 1 (perfect consistency). The minimum basis on which a necessary (sufficient) relation can be claimed is 0.90 (0.75) [29, 30].

Coverage by contrast, measures empirical relevance [28, 29]. For necessity, coverage measures the frequency with which an outcome occurs relative to a causal condition. Here very low coverage scores indicate that a causal condition is present in almost all cases, regardless of whether they display the outcome. In this scenario, a necessary condition would be deemed trivial. For sufficiency, coverage indicates the degree to which a condition (or combination of conditions) explains all occurrences of an outcome. If coverage scores are very low this would indicate that the causal condition explains only a limited set of the cases with an outcome. Coverage scores are also calculated by the fsQCA software and range from 0 (no coverage) to 1 (full coverage). It is suggested that when testing for necessity, coverage scores should not be lower than 0.5 and that no cause should be deemed necessary, independent of a theory that recognizes it as a relevant cause [29]. Minimum coverage scores are not suggested for sufficient relations since configurational methods recognize that an outcome may be produced via different combinations of conditions.

The final fsQCA stage involves a process of ‘logical reduction’ where a simplified statement is made about which conditions are necessary/sufficient for an outcome (termed a solution path). In a conventional QCA, this is achieved through Boolean Algebra. For example, if two combinations of conditions are found to be sufficient, one with causal conditions A, B, and C and the other with causal conditions A and B (but not C), we could reduce this to one configuration: AB, since the outcome occurs whether condition C is present or absent. In fsQCA, an equivalent process is undertaken by the software using the Quine-McCluskey algorithm. This algorithm takes into account the more complex features of fsQCA, including consistency scores [21].

Overall consistency and coverage scores are used to describe the logically reduced solution paths. These measures are a calculation of how well an outcome is explained when all of the reduced solution paths are considered. Generally speaking, overall consistency is an average of the consistency scores of each of the individual solution paths found for an outcome. Overall coverage is a measure of how well the cases displaying an outcome are covered by the logically reduced solution paths.

### Health outcomes

This study examines two outcomes: adult female mortality rates (AFM) and infant mortality rates (IMR). The former was chosen since most T&C workers are female. The latter was chosen for its rapid response and sensitivity to macro-level policy changes [31–33]. IMR was conceptualized to have been potentially impacted both directly, through T&C workers having children, and indirectly, if the phase-out influenced health important conditions at the national-level. Two national-level conditions highlighted by the EMCONET framework are material deprivation and economic inequality [14]. Both of these conditions may have been impacted through shifts in T&C employment (e.g., through additional provision or loss of wages) and both have been previously associated with IMR (e.g., [34, 35]).

There is a relatively robust body of literature which finds evidence for changes in national-level health outcomes following changing macro-economic conditions [36–39]. Of particular relevance here is the evidence for the health impacts of job loss (e.g., [39]). While fewer studies have measured the health effect of employment growth [40], there are many pathways through which we can expect it to impact health at the national-level [9]. Furthermore, although much of the public health literature surrounding changing macroeconomic conditions is focused on the developed world, evidence suggests that such changes also have important implications for national-levels of health in poorer countries [41, 42], where much of the T&C sector is concentrated.

AFM and IMR were obtained from Rajaratnam and colleagues [43, 44]. Historically, the usefulness of adult mortality data has been hindered by a range of well-known weaknesses [45, 46]. Models have often extrapolated adult mortality from child mortality. Ambiguity in both the sources of data and the methods used has also hindered replication of results [46]. Documenting short-term fluctuations and linking them to changing socio-economic contexts requires far greater detail than past methods have provided [47].

The authors of the data this study utilizes, by contrast, estimate AFM (IMR) through a variety of sources including vital registration systems, sample registration systems, and nationally representative survey/census data [43, 44]. These methods demonstrate a higher predictive validity and are transparent and replicable [43, 44, 46]. Moreover, the authors specifically acknowledge that a main advantage of their data is that it can be linked to changes in socio-economic contexts. AFM is summarized by the probability that an individual who is 15 years old will die before age 60. IMR is summarized by the probability of death before age 1, conditional on surviving to 1 month.

### Case selection

Countries were included in this analysis if, between 2000 and 2004, employment in the T&C sector (as a proportion of total manufacturing employment) was greater than 10 %, given that more than 10 % of the working population was employed in manufacturing. Total manufacturing and T&C employment figures were obtained from the United Nations Industrial Development Organization (UNIDO) [20]. Data on the proportion of the working population employed in industry were obtained from the World Bank [48]. While 53 countries were initially identified as reliant on the sector (Table 1), only 32 countries were ultimately used for the analysis (Table 2). Inclusion of countries was limited by the quality of mortality data and the availability of data used to operationalize the causal conditions (Table 3). Countries were excluded if mortality data was characterized by relatively high and/or erratic levels of uncertainty. While excluded countries were comprised of both highly developed and less developed countries, it is unclear how their inclusion might have impacted the results of the analyses. This work thus reiterates calls for better quality cross-national health and social policy data. Despite this limitation, the number of cases included in this study well exceeds the minimum number of cases below which there is a high chance that a fsQCA will find an association due to random variation [49].

**Table 1:** Countries identified for inclusion

Country	Year	T & C employment combined as % of total manufacturing employment^a^	% of working population in industry^c^
Albania	2004	26.76	13.6
Azerbaijan	2004	12.92	11.9
Bangladesh^b^	2004	40	13.7
Bolivia	2001	14.81	20.5
Botswana	2004	33.41	22.6
Brazil	2004	12.76	21
Bulgaria	2004	29.53	32.9
Cambodia	2000	73.60	10.5
China	2004	18.11	22.5
China, Hong Kong SAR	2004	27.90	15.6
China, Macao SAR	2004	82.70	25.2
Colombia	2004	22.30	19.9
Costa Rica	2003	17.60	22
Croatia	2004	13.78	29.8
Ecuador	2004	10.87	17.5
Egypt	2004	28.51	20
Estonia	2004	17.98	34.9
Greece	2004	13.73	22.5
Guatemala	2004	40.57	19.5
Hungary	2004	10.19	32.8
India	2004	21.03	16.1
Indonesia	2004	22.90	18
Italy	2004	11.14	30.8
Jamaica	2004	16.28	18.3
Jordan	2004	15.49	21.8
Kuwait	2001	16.75	18.3
Korea	2004	10.04	27.5
Kyrgyz Republic	2004	11.48	17.6
Latvia	2004	13.74	27.3
Lithuania	2004	21.97	28.2
Mauritius	2004	66.83	33.5
Mexico	2003	14.42	24.8
Mongolia	2004	51.23	16.1
Morocco	2004	41.53	19.5
Nepal	2002	28.28	13.4
Peru	2004	35.60	41.7
Philippines	2003	18.54	15.8
Poland	2004	10.24	28.8
Portugal	2004	24.24	31
Puerto Rico	2000	18.42	19.4
Qatar	2004	21.51	41
Republic of Macedonia	2004	36.42	32.8
Romania	2004	24.29	31.2
Saudi Arabia	2003	14.75	21
Serbia & Montenegro	2001	17.82	26.9
Slovakia	2004	10.56	39
South Africa	2004	12.82	26.1
Sri Lanka	2001	49.32	24.1
Syrian Arab Republic	2004	25.41	25.6
Thailand	2002	18.04	20.5
Turkey	2004	34.55	23
Uruguay	2004	13.14	21.4
Vietnam	2004	23.04	17.4

Sources: United Nations Industrial Development Organization (2011): INDSTAT2, Industrial Statistics Database (Edition: 2011). ESDS International, University of Manchester. doi: 10.5257/unido/indstat2/2011. Industrial Statistics Database 2011 at the 2-digit level of ISIC Code (Revision 3).

^a^ISIC codes 17 for textiles, 18 for wearing apparel and D for total manufacturing

^b^Since data was not available on Bangladesh in the UNIDO database, and because the country is oft cited as extremely reliant on the textile and clothing sector, data for Bangladesh taken from the IMF Working Paper by Mlachila and Yang (2004): The End of Textiles Quotas: A Case Study of the Impact on The End of Textiles Quotas on Bangladesh

^c^World Bank (2011): World Development Indicators (Edition: September 2011). ESDS International, University of Manchester. doi:http://dx.doi.org/10.5257/wb/wdi/2011-09

**Table 2:** Final set of included countries

Azerbaijan^a^	Hungary^b^	Peru
Bangladesh	India	Philippines
Brazil	Indonesia^a^	Poland
Bulgaria	Italy	Portugal
China	Korea	Romania^b^
Colombia	Kyrgyz Republic	Slovakia
Croatia	Latvia	South Africa
Ecuador	Lithuania	Sri Lanka^a^
Egypt^a^	Mauritius	Thailand
Estonia^b^	Mexico	Turkey
Greece	Morocco^a^	

^a^only for IMR

^b^only for AFM

**Table 3:** Excluded countries and reasons for exclusion

Country	Reason for exclusion
Albania	AFM; IMR
Azerbaijan^a^	AFM
Bolivia	ILO ISI, UNIDO
Botswana	ILO ISI
Cambodia	ILO ISI, UNIDO
China, Hong Kong SAR	ILO ISI
Costa Rica	UNIDO
China, Macao SAR	ILO ISI
Egypt^a^	AFM
Estonia^b^	IMR
Hungary^b^	IMR
Guatemala	ILO ISI
Indonesia^a^	AFM
Jamaica	ILO ISI
Jordan	ILO ISI
Kuwait	ILO ISI: UNIDO
Mongolia	ILO ISI
Morocco^a^	AFM
Nepal	AFM; IMR
Puerto Rico	ILO ISI; UNIDO
Qatar	ILO ISI
Republic of Macedonia	AFM; IMR
Saudi Arabia	ILO ISI
Serbia and Montenegro	ILO ISI
Sri Lanka^a^	AFM
Syrian Arab Republic	ILO ISI
Uruguay	ILO ISI
Vietnam	ILO ISI

*AFM* Adult female mortality data unsuitable, *IMR* Infant mortality data unsuitable, *UNIDO* Employment figures not available from UNIDO, *ILO ISI* country not included in ILO Income Security Index

^a^country included for infant mortality

^b^country included for adult female mortality

### Causal conditions

Five causal conditions were selected for inclusion in the fsQCA: countries’ level of development; (2) labour market protection; (3) welfare state protection; and (4) T&C employment loss or (5) growth after the phase-out. There are a variety of approaches that can be used to select causal conditions for a fsQCA [50, 51]. Here conditions were selected in direct response to the research question. A development indicator was included to contextualize how employment changes impacted health in countries of different levels of development and to group countries with similar health profiles together. Since the expectation is that the chosen causal conditions will combine in different ways to impact the health, they can also be seen as selected via the *conjunctural* approach [50, 51]. This approach is described in QCA literature as best aligned with the characteristics of a fsQCA analysis [50]. Specific hypotheses concerning these conditions were not made since the nature of this study tends towards theory development rather than theory testing.

### Fuzzy-set membership scores

Fuzzy-set membership scores are assigned through a process called calibration [21]. Calibration refers to the transformation of outcome indicators and causal conditions into membership sets. This procedure requires the use of theoretical and substantive knowledge to denote meaningful differences in the data in order to define cases’ degree of membership in the set created by an indicator. Calibration methods can be either direct or indirect. In the direct method, three thresholds are specified which correspond to the qualitative breakpoints of full membership (1), the cross-over point (.5), and full non-membership (0). At the crossover point there is maximum ambiguity of whether a case is more “in” or “out” of a set. Once these breakpoints are specified, fuzzy membership scores are assigned by the fsQCA software. Generally speaking, the software calculates scores by translating variable scores into the metric of log odds [21]. A strength of this method is that it is able to calculate precise fuzzy-set scores when there is similarly precise variation in the data.

The indirect method, by contrast, relies on a broad grouping of cases into a number of categories which represent different degrees of membership. This method is generally used when it is difficult to translate data using the three qualitative breakpoints or when the data is better aligned with a smaller number of membership categories (e.g., when there is less precise variation in the data).

In this study, the direct method was used to transform the health outcome indicators, countries’ level of development and employment growth and loss after the MFA phase-out. This is because data associated with these conditions could be anchored to the three qualitative breakpoints and because using the direct calibration resulted in more precise fuzzy-set scores. An indirect calibration method was used to transform the causal conditions of countries’ labour market and welfare state protection. This is because the data used to operationalize these conditions was not aligned with the direct calibration method and better transformed through the indirect method, as will be made clearer below.

External standards with which to calibrate the conditions included in this study do not yet exist. As a consequence, calibration thresholds were established based on the structure of the data and careful considerations of what meaningful thresholds would require in terms of best representing the condition. Sensitivity analyses were carried out which evaluated the impact of lower and higher thresholds and demonstrated little difference in fuzzy-set scores and final results. Further details of the calibration process for each of the conditions are noted below. The raw data and corresponding fuzzy-set scores for each of the outcomes and causal conditions can be found in an additional file (Additional file 1).

For each outcome indicator, AFM and IMR, a ‘Health Improving’ and ‘Health Worsening’ membership set was constructed. Relative changes in mortality rates were calculated based on the five-year period preceding (2000–2004) and following the phase-out (2005–2009). Data for these calculations are displayed in Tables 4 and 5. Although this is a relatively short time to examine changes in population health, it is consistent with studies which show an association between unemployment and adult mortality after a similar time lag [41, 52–56]. In relation to FMR (IMR), the qualitative breakpoints for the health improving set were conceptualized respectively as a 3 % (4 %) increase in mortality rate reduction, a 0 % change in mortality rate reduction and a 3 % (4 %) decrease in mortality rate reductions. Scores in the sets of ‘health worsening’ were taken to be the negation of health improving scores and calculated by subtracting a country’s score in the health improving set from 1. In terms of AFM, 10 of the 27 analyzed countries experienced health improvement after the MFA phase-out. In terms of IMR, 17 of the 29 analyzed countries experienced health improvement.

**Table 4:** Relative changes in adult female mortality rates

Country	Adult female mortality rate (per 1000)				Percent change in adult female mortality reduction		Difference between pre & post MFA periods
	2000	2004	2005	2009	Pre MFA (2000–2004)	Post MFA (2005–2009)	
Bangladesh	135.6	126.1	124.8	121.4	7.01	2.72	−4.28
Brazil	122.4	119	117.9	111.4	2.78	5.51	2.74
Bulgaria	97.8	91.8	92	88.5	6.13	3.80	−2.33
China	118.6	105.4	102.5	92.5	11.13	9.76	−1.37
Colombia	88.7	78.7	76.2	68.6	11.27	9.97	−1.30
Croatia	74.9	66.1	64.9	62.9	11.75	3.08	−8.67
Ecuador	98.6	90.7	88.7	80.6	8.01	9.13	1.12
Estonia	119.2	102	98.7	87.6	14.43	11.25	−3.18
Greece	48.4	46.5	45.8	42.4	3.93	7.42	3.50
Hungary	115	107.7	106.6	104	6.35	2.44	−3.91
India	188.6	166.4	161.7	147.3	11.77	8.91	−2.87
Italy	50.5	45	44.2	42	10.89	4.98	−5.91
Korea	61.7	51.2	49.1	42	17.02	14.46	−2.56
Kyrgyz Republic	154.7	146	145.6	144.4	5.62	0.82	−4.80
Latvia	120.9	116.7	117.9	120	3.47	−1.78	−5.26
Lithuania	105.1	104.5	108.5	115.5	0.57	−6.45	−7.02
Mauritius	110	108.5	108.1	106.6	1.36	1.39	0.02
Mexico	101.4	95.9	94.7	89.8	5.42	5.17	−0.25
Peru	97.6	95	94.1	82.9	2.66	11.90	9.24
Philippines	118.8	120.1	119.8	116	−1.09	3.17	4.27
Poland	86.3	78.3	78.8	78.3	9.27	0.63	−8.64
Portugal	66.4	59.1	56.8	50.2	10.99	11.62	0.63
Romania	108.3	100.4	98.5	91	7.29	7.61	0.32
Slovak Republic	81.8	77.9	77.4	75.2	4.77	2.84	−1.93
South Africa	316.5	430.2	444.2	450	−35.92	−1.31	34.62
Thailand	124.9	117.2	114.1	102.7	6.16	9.99	3.83
Turkey	102.7	92.8	91.2	86.1	9.64	5.59	−4.05

**Table 5:** Relative changes in infant mortality rates

Country	Infant mortality rate (per1000)				Percent change in infant mortality reduction		Difference between pre and post MFA periods
	2000	2004	2005	2009	Pre MFA (2000–2004)	Post MFA (2005–2009)	
Azerbaijan	24.5	19.1	17.8	13.2	22.14	25.84	3.71
Bangladesh	23.2	18.7	17.8	14.8	19.18	16.72	−2.46
Brazil	13.3	10.9	10.4	8.6	18.23	17.71	−0.52
Bulgaria	7.2	5.5	5.2	4.3	23.86	17.48	−6.38
China	9.5	6.8	6.2	4.8	28.87	21.56	−7.32
Colombia	8.9	7.3	6.9	5.3	18.39	22.30	3.92
Croatia	2.1	1.8	1.7	1.3	15.96	21.76	5.80
Ecuador	16.8	14.3	13.7	11.5	14.97	16.34	1.37
Egypt	15.8	11.8	10.9	8.1	25.47	26.01	0.53
Greece	1.9	1.5	1.5	1.1	19.68	26.90	7.22
India	22.4	19.4	18.8	16.4	13.06	12.66	−0.40
Indonesia	17.2	15.1	14.7	12.9	11.89	12.19	0.31
Italy	1.5	0.9	0.9	0.8	35.17	13.33	−21.84
Korea	2.7	2.4	2.3	1.8	10.00	23.58	13.58
Kyrgyz Republic	21.1	19.4	19.0	17.0	8.20	10.22	2.03
Latvia	4.8	3.7	3.5	3.2	22.96	7.98	−14.99
Lithuania	3.9	3.3	3.2	2.6	16.20	16.51	0.31
Mauritius	5.0	3.7	3.5	3.1	26.89	11.11	−15.78
Mexico	12.9	10.8	10.3	8.6	16.73	16.75	0.02
Morocco	16.0	12.9	12.2	9.8	19.16	19.89	0.72
Peru	13.9	11.1	10.5	8.6	20.60	18.13	−2.47
Philippines	11.7	10.5	10.2	8.8	10.26	12.91	2.65
Poland	2.5	2.0	1.9	1.7	22.05	13.16	−8.89
Portugal	2.5	1.4	1.3	1.0	41.94	22.05	−19.89
Slovak Republic	3.6	3.1	3.0	2.3	13.65	21.96	8.31
South Africa	16.2	23.1	25.1	24.0	−42.44	4.30	46.75
Sri Lanka	4.3	3.0	2.8	2.3	29.37	19.08	−10.29
Thailand	3.1	2.5	2.3	1.9	19.61	19.74	0.13
Turkey	14.9	12.7	12.3	10.3	14.58	16.71	2.12

The United Nation’s Human Development Index (HDI) was used to assign scores in the set of ‘Highly Developed Countries’ [57]. This data reflects conditions in countries in 2004. Data was directly calibrated in a way which aligned with the Index’s rating of countries into the categories of High, Medium and Low Human Development. The qualitative breakpoints were conceptualized as 0.9, 0.8 and 0.5, respectively. The cross-over point was chosen at 0.8 since below this point, countries are deemed as having medium human development. Countries receiving a HDI score of lower than 0.5 are deemed by the Index as having Low Human Development.

Countries’ labour market protection was indirectly calibrated based on the number of Fundamental ILO Conventions ratified by a country [58]. Here a six-value fuzzy-set [29] was used to assign scores in the set of ‘Protective Labour Market Policies, taking into account the number of Conventions ratified before the MFA phase-out, as well as additional ratifications made prior to 2009. Table 6 further demonstrates this calibration process. Since these Conventions represent minimum standards, relatively strict thresholds were set for countries to be characterized as having protective policies.

**Table 6:** Scoring procedure for protective labour market policies

Number of fundamental ILO conventions ratified in 2004	Initial fuzzy set score	Score adjustment for additional ratifications by 2009
8	1	NA
7	0.6	+0.2
6	0.4	+0.2-0.4
4–5	0.2	+0-0.6
3 or less	0	+0-0.8

Welfare state protection was measured and calibrated using the ILO Income Security Index [59]. This Index uses a range of input, process and outcome indicators and categorizes countries into one of four clusters. ‘Pacesetting’ countries are characterized as scoring highly across all indicators. ‘Conventional’ countries score highly only on input and process indicators. ‘Pragmatists’ score high on outcome indicators and ‘Much-to-be-done’ countries score low across all indicators. These categorizations were used to assign scores in the set of ‘Protective Welfare State Policies’ since they delineate important qualitative features of countries. Another option would have been to use the individual index scores to directly calibrate fuzzy-set memberships; however, index scores do not directly align with the qualitative clusters. For instance, a Conventional country might score lower on the Index than a Much-To-Be-Done country. Using the direct calibration method thus would have clouded important qualitative differences between countries. For this reason, scores were indirectly assigned as follows: Pacesetters (1), Conventionals (.67), Pragmatists (.33), and Much-To-Be-Dones (0).

A direct calibration method was used to assign fuzzy-set scores in the employment growth and loss membership sets. Qualitative thresholds were chosen with a consideration of the variation of change across countries and with a consideration that changes would need to be somewhat significant to influence health at the population level. Employment growth and loss were treated separately, rather than as a single employment change condition, since the qualitative breakpoints of a single membership set were tasked with meeting two conditions found to be at odds with each other. Specifically, a single membership set would need both to differentiate between countries experiencing employment growth and loss (conditions which have different implications for health) and denote meaningful changes in employment (i.e., changes that would have feasibly made an impact on population health at the national level). For a single membership set to differentiate between countries experiencing employment growth and loss, the cross-over point (of 0.5) would need to be set at a 0 % change in employment. However, this would mean that countries experiencing a small change in employment, for example a 5 % increase, would be characterized as largely ‘in the set’ of employment change. This was seen as problematic since small changes are unlikely to result in discernable changes in national levels of health. Using two membership sets however, allowed for meaningful changes in employment to be more accurately accounted for. This is because the cross-over point for each of these sets could be set at a 5 % change in employment loss/growth. In this way, countries with a small change in employment are characterized as only somewhat in the membership set. Fuzzy-set scores were therefore calibrated across two membership sets based on percent changes in T&C employment between 2004 and 2008 (or the closest year for which data was available). For the Employment Growth (Loss) membership set, the qualitative breakpoints were conceptualized at a 15 % increase (decrease), a 5 % increase (decrease), and 0 % increase (decrease). Employment figures were obtained from UNIDO [20].

Tables 7 and 8 respectively summarize the fuzzy-set scores for the AFM and IMR membership sets, as well as for the five causal conditions. These tables demonstrate ample variation between countries both in terms of the outcomes and causal conditions.

**Table 7:** Fuzzy-set data matrix for adult female mortality

Country	Highly developed	Protective labour market polices	Protective welfare state policies	Employment growth	Employment loss	Improving AFM	Worsening AFM
Azerbaijan	0.35	1	0	0	0.99	NA	NA
Bangladesh	0.06	0.6	0	1	0	0.01	0.99
Brazil	0.48	0.6	0.67	0.89	0	0.94	0.06
Bulgaria	0.62	1	0.67	0	0.98	0.09	0.91
China	0.42	0.2	0	0.98	0	0.2	0.8
Colombia	0.48	0.8	0	0	0.36	0.21	0.79
Croatia	0.8	1	0.67	0	0.98	0	1
Ecuador	0.41	1	0.67	0.24	0.01	0.75	0.25
Egypt	0.27	1	0	0.02	0.11	NA	NA
Estonia	0.85	1	0.33	0	1	0.04	0.96
Greece	0.97	1	0.33	0.01	0.29	0.97	0.03
Hungary	0.89	1	0.33	0	1	0.02	0.98
India	0.13	0.2	0	0.99	0	0.05	0.95
Indonesia	0.29	1	0	0.75	0	NA	NA
Italy	0.99	1	0.33	0	0.92	0	1
Korea	0.97	0.2	0.33	0	0.9	0.07	0.93
Kyrgyzstan	0.28	1	0	0	1	0.01	0.99
Latvia	0.79	0.8	1	0	1	0.01	0.99
Lithuania	0.85	1	0.33	0	1	0	1
Mauritius	0.5	0.8	0.67	0	0.95	0.51	0.49
Mexico	0.65	0.4	0.67	0	1	0.44	0.56
Morocco	0.17	0.6	0	0	0.73	NA	NA
Peru	0.42	1	0	0	0.97	1	0
Philippines	0.41	0.8	0.67	0	0.63	0.99	0.01
Poland	0.87	1	1	0	0.74	0	1
Portugal	0.96	1	1	0	0.89	0.65	0.35
Romania	0.54	1	0.67	0	1	0.58	0.42
Slovakia	0.84	1	1	0	0.99	0.13	0.87
South Africa	0.19	1	0.67	0	1	1	0
Sri Lanka	0.39	1	0.67	1	0	NA	NA
Thailand	0.46	0.2	0	0.71	0	0.98	0.02
Turkey	0.39	1	0.67	0.04	0.06	0.02	0.98

**Table 8:** Fuzzy-set data matrix for infant mortality

Country	Highly developed	Protective labour market polices	Protective welfare state policies	Employment growth	Employment loss	Improving IMR	Worsening IMR
Azerbaijan	0.35	1	0	0	0.99	0.94	0.06
Bangladesh	0.06	0.6	0	1	0	0.14	0.86
Brazil	0.48	0.6	0.67	0.89	0	0.4	0.6
Bulgaria	0.62	1	0.67	0	0.98	0.01	0.99
China	0.42	0.2	0	0.98	0	0	1
Colombia	0.48	0.8	0	0	0.36	0.95	0.05
Croatia	0.8	1	0.67	0	0.98	0.99	0.01
Ecuador	0.41	1	0.67	0.24	0.01	0.74	0.26
Egypt	0.27	1	0	0.02	0.11	0.6	0.4
Estonia	0.85	1	0.33	0	1	NA	NA
Greece	0.97	1	0.33	0.01	0.29	1	0
Hungary	0.89	1	0.33	0	1	NA	NA
India	0.13	0.2	0	0.99	0	0.43	0.57
Indonesia	0.29	1	0	0.75	0	0.56	0.44
Italy	0.99	1	0.33	0	0.92	0	1
Korea	0.97	0.2	0.33	0	0.9	1	0
Kyrgyzstan	0.28	1	0	0	1	0.82	0.18
Latvia	0.79	0.8	1	0	1	0	1
Lithuania	0.85	1	0.33	0	1	0.56	0.44
Mauritius	0.5	0.8	0.67	0	0.95	0	1
Mexico	0.65	0.4	0.67	0	1	0.5	0.5
Morocco	0.17	0.6	0	0	0.73	0.63	0.37
Peru	0.42	1	0	0	0.97	0.14	0.86
Philippines	0.41	0.8	0.67	0	0.63	0.88	0.12
Poland	0.87	1	1	0	0.74	0	1
Portugal	0.96	1	1	0	0.89	0	1
Romania	0.54	1	0.67	0	1	NA	NA
Slovakia	0.84	1	1	0	0.99	1	0
South Africa	0.19	1	0.67	0	1	1	0
Sri Lanka	0.39	1	0.67	1	0	0	1
Thailand	0.46	0.2	0	0.71	0	0.53	0.47
Turkey	0.39	1	0.67	0.04	0.06	0.83	0.17

### Process tracing

Ideally, all cases included in an fsQCA would be studied in-depth; however, for this study this would require a prohibitively large number of studies. Therefore, twelve countries were selected for in-depth analysis so that each of the fsQCA solutions could be explored by at least one typical case (i.e., one that is characterized both by the configuration and outcome of the necessary/sufficient relation). When a fsQCA solution was characterized by multiple typical cases, a comparative approach was undertaken since our confidence in a causal mechanism is increased if it is found to be in place across multiple typical cases [26]. A comparative study design was also undertaken to take advantage of deviant cases. These cases are members of a configuration characterized by a logically reduced solution, but are not members of the associated outcome. As such, these cases provide evidence against a necessary/sufficient relation but represent a potentially useful opportunity to understand the fsQCA results. For example, the most likely reason for a deviant case in a sufficient relation is the omission of a causal condition of which the deviant case is not a member but the typical cases are [26].

In line with process tracing literature [27], evidence was collected to build a narrative about the overall structure of each country’s T&C sector (e.g., its workers, how employment changed after the phase-out, alternative employment opportunities) and its labour market and welfare state policies. Next, the aim was to inductively work backwards in search of a plausible causal mechanism that might help explain the fsQCA results.

Because selection bias is particularly acute in process tracing research [27], an attempt was made to minimize this bias by using a systematic process to search for evidence. A preliminary search strategy found that traditional databases, such as the Applied Social Science Index and Abstracts database, returned a dearth of relevant material; therefore, Google and Google Scholar were used to locate sources of evidence. Search keywords included the country name, ‘Multi-Fibre Arrangement, ‘health’, ‘employment’, ‘textile and clothing sector’, ‘apparel’, and ‘garments’. Once a narrative of a country’s T&C sector was constructed, material regarding labour market and social policies was searched for, particularly across international organizations including the International Labour Organization, World Bank and Asian Development Bank.

## Results

### FSQCA results

**Fig. 3:**
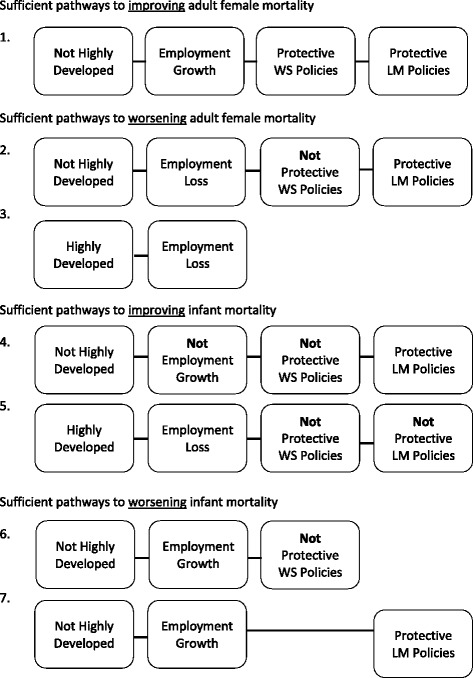
FsQCA solution paths

Necessity and sufficiency analyses were carried out with fsQCA software. No condition was found to be necessary. The truth table displayed in Table 9 indicates the results of the sufficiency analyses. The final logically reduced configurations, deemed solution paths, are displayed in Table 10 and Fig. 3. Seven solution paths are found which relate changes in employment after the phase-out to either changing AFM or IMR. Two additional solution paths are displayed (italicized) in Table 10 but are not further explored since they are characterized neither by employment growth nor loss.

**Table 9:** Results of sufficiency analyses

	Adult female mortality			Infant mortality		
Configuration	Cases	Health improving consistency	Health worsening consistency	Cases	Health improving consistency	Health worsening consistency
h*M*W*G*l	Brazil	0.975*	0.432	Brazil, Sri Lanka	0.486	0.993*
h*m*w*G*l	China, India, Thailand	0.426	0.702	China, India, *Thailand*	0.540	0.887*
h*M*w*G*l	Bangladesh	0.582	0.753*	Bangladesh, *Indonesia*	0.604	0.903*
h*M*W*g*L	Philippines, South Africa	0.705	0.693	Philippines, South Africa	0.642	0.560
h*M*w*g*L	Kyrgyz Republic, Peru	0.613	0.720	Azerbaijan, Kyrgyz Republic, Morocco, *Peru*	0.787*	0.523
h*M*W*g*l	Ecuador, Turkey	0.629	0.629	Ecuador, Turkey	0.881*	0.455
h*M*w*g*l	Colombia	0.681	0.676	Colombia, Egypt	0.933*	0.603
H*M*W*g*L	Bulgaria, Croatia, Latvia, Poland, *Portugal*, Romania, Slovak Republic	0.398	0.821*	Bulgaria, Croatia, Latvia, Poland, Portugal, Slovak Republic	0.469	0.660
H*m*W*g*L	Mexico	0.555	0.867*	Mexico	0.673	0.667
H*M*w*g*L	Estonia, Hungary, Italy, Lithuania	0.416	0.816*	Italy, Lithuania	0.685	0.606
H*m*w*g*L	Korea	0.578	0.894*	Korea	0.887*	0.492
H*M*w*g*l	Greece	0.745	0.532	Greece	0.954*	0.526

Italicized countries represent deviant cases, they are members of the sufficient configuration but not of the outcome

*H* highly developed; *M* protective labour market; *W* protective welfare state; *G* employment growth; *L* employment loss (lower case signifies the negation of these conditions)

*Consistency greater than 0.75

**Table 10:** Logical reduction results

Adult female mortality	Cases	Solution path consistency	Solution path coverage	Overall coverage	Overall consistency
Health improving					
1. h*M*W*G*l	Brazil	0.975	0.082	0.082	0.975
Health worsening					
2. H*g*L	Italy, Korea, Hungary, Estonia, Lithuania, Slovak Republic, Croatia, Latvia, Poland, Bulgaria	0.841	0.621	0.697	0.830
3. h*M*w* G*l	Bangladesh	0.753	0.079	0.697	0.830
Infant mortality					
Health improving					
M*w*g*l	Colombia, Egypt, Greece	0.897	0.267	0.536	0.826
h*M*g*l	Ecuador, Turkey Colombia, Egypt	0.904	0.251	0.536	0.826
4. h*M*w*g	Kyrgyz Republic, Colombia, Azerbaijan, Morocco, Egypt	0.824	0.400	0.536	0.826
5. H*m*w*g*L	Korea	0.887	0.107	0.536	0.826
Health worsening					
6. h*w*G*l	Bangladesh, India, Indonesia, China, Thailand	0.842	0.270	0.303	0.856
7. h*M*G*l	Indonesia, Sri Lanka, Bangladesh, Brazil	0.916	0.214	0.303	0.856

*H* highly developed, *M* protective labour market, *W* protective welfare state, *G* employment growth, *L* employment loss (lower case signifies the negation of these conditions)

In terms of AFM, logical reduction resulted in only one solution path to health improvement. This first solution path concerns countries which are not highly developed and relates increases in T&C employment to an improvement of AFM in the context of protective labour market and welfare state policies. This solution’s overall coverage however, at 0.082, is very low (the only case characterized by this solution path is Brazil), indicating that there are many other cases exhibiting an improvement in AFM which are not covered by this solution.

In terms of worsening AFM, the logical reduction process resulted in two solution paths. The first (solution path 2) concerns highly developed countries and relates decreases in T&C employment to a worsening of AFM, regardless of the presence or absence of protective labour market and welfare state policies. The next solution (solution path 3) concerns countries which are not highly developed. It relates increases in T&C employment to a worsening of AFM in the presence of protective labour market (but not welfare state) policies. While the first of these solution paths covers ten countries, Bangladesh is the only country characterized by the second. Together however, the two configurations have a relatively high overall solution coverage of 0.697. This means that the majority of countries exhibiting a worsening of AFM are covered by these solution paths.

In terms of IMR, logical reduction resulted in four solution paths to health improvement. Again, two of these configurations are of less interest to the objective of this work, since they are characterized by neither employment growth nor loss. Of the remaining solution paths, one (solution path 4) concerns not highly developed countries and relates an improvement of IMR to either T&C employment loss or no change in T&C employment, in the context of protective labour market (but not welfare state) policies. The next solution path (solution path 5) relates employment loss in highly developed countries to an improvement of IMR in the context of lacking protective labour market and welfare state policies. The overall coverage score of these solutions paths is 0.536 which indicates that they cover about half of the countries exhibiting this outcome.

In terms of worsening IMR, logical reduction resulted in two solution paths. The first (solution path 6), relates employment growth in not highly developed countries to a worsening of IMR in the context of lacking protective welfare state policies, regardless of the presence or absence of protective labour market policies. The second (solution path 7), relates employment growth in not highly developed countries to a worsening of IMR in the context of protective labour market policies, regardless of the presence or absence of protective welfare state policies. Together, these two solution paths have an overall low coverage of 0.303, indicating that there are many other cases exhibiting a worsening of IMR that are not covered by these solution paths.

### In-depth country studies

As previously mentioned, twelve in-depth country studies (Table 11) were undertaken to further investigate the fsQCA results. Across all countries studied in-depth, evidence was found which confirmed the assumption that females represent the majority of T&C workers. Females in these countries were found to represent anywhere from 70 to 90 % of the total T&C workforce.

**Table 11:** Country case-studies

Solution path	Outcome	Countries
1. h*M*W*G*l	Improving AFM	Brazil
2. H*g*L	Worsening AFM	Italy, Slovak Republic, Korea, Portugal (deviant)
3. h*M*w*G*l	Worsening AFM	Bangladesh
4. h*M*w*g	Improving IMR	Kyrgyz Republic, Peru (deviant)
5. H*m*w*g*L	Improving IMR	Korea
6. h*w*G*l	Worsening IMR	China, Thailand (deviant)
7. h*M*G*l	Worsening IMR	Sri Lanka, Bangladesh, Indonesia (deviant)

*H* highly developed, *M* protective labour market, *W* protective welfare state, *G* employment growth, *L* employment loss (lower case signifies the negation of these conditions)

Brazil was investigated in the first solution path which relates employment growth in not highly developed countries to AFM improvement in the context of protective labour market and welfare state policies. Here process tracing did not lead to the construction of a potential causal mechanism. This is because it was found that a conditional cash transfer programme (‘Bolsa Familia’) was introduced in Brazil in 2003 which was likely to have influenced positively on AFM after the phase-out. By 2006, this programme covered 11 million households and has since been noted for its role in reducing poverty and inequality [60]. It has also been associated with reductions in childhood mortality [61].

In the second solution path, four countries were investigated: Italy, the Slovak Republic, Korea and Portugal (as a deviant case). This solution relates decreases in T&C employment in highly developed countries to a worsening of AFM regardless of the presence or absence of protective labour market and welfare state policies. This finding is puzzling because we might expect protective policies to act as a buffer to the potentially negative impacts of employment loss. Here process tracing efforts were able to uncover a potential causal mechanism. Across the typical cases, evidence was found which suggests that regardless of whether a country could be characterized by protective policies, T&C workers losing their employment would have had 1) few alternative employment opportunities and 2) little access to social protection.

In Italy, this latter point is related to labour regulations which exempt employees from important protective social policies when they are employed in firms of 15 employees or less [62, 63]: precisely the type of firms where T&C workers were likely to have lost their employment after the phase-out [64–66]. It is also worth noting that working conditions after the MFA phase-out were likely to have worsened in Italy, for those remaining employed in the sector [66]. In the Slovak Republic, evidence suggests that T&C workers losing their employment were likely to have been employed under work arrangements which were introduced during deregulation of the country’s labour code in 2001 [67]. These arrangements exist outside of formal employment relationships and preclude workers from important protections such as unemployment insurance [68, 69]. In regards to Korea, evidence suggests that female T&C workers are overrepresented in nonstandard and irregular jobs [70–73] and as such are likely unable or unwilling to make contributions towards social insurance schemes [74–76].

As a deviant case, Portugal did not experience a worsening of AFM. Here evidence was found which suggests that T&C workers had greater access to social protection, since their work was likely to be characterized by a more standard employment relationship [77, 78]. In addition to unemployment insurance, Portuguese T&C workers losing their employment would have likely been covered by the country’s collective dismissal regulations. These regulations require employers to give workers advance notice of dismissals and paid time off to look for alternative work. Workers are also entitled to severance pay, possible re-training opportunities and/or early retirement [63].

The third solution path was characterized by only one country: Bangladesh. This solution describes a sufficient relationship between AFM worsening and employment growth in less developed countries with protective labour market (but not welfare state) policies. Process tracing efforts undertaken in regards to this solution found that the T&C sector in Bangladesh is characterized by a range of complex and often contradictory processes [79]. This relates primarily to the type of firms within which women work and the different spheres of their lives which are impacted, sometimes negatively, sometimes positively, by work in the sector. Because evidence suggests that Bangladeshi T&C workers have little access to social protection and that employment growth after the MFA phase-out likely took place in firms with poor (and worsening) working conditions [80, 81] ultimately a potential causal mechanism emerges which directs attention to these conditions.

The final four solution paths concern changes in IMR. Here process tracing efforts were unable to uncover potential causal mechanisms. In some cases this related to a lack of evidence on whether T&C workers were having children. In others, this related to the impact of a large Tsunami which struck Southeast Asia in late December 2004. Indirect pathways were also explored; however, evidence was not uncovered which could link either material deprivation or economic inequality to the phase-out and changing IMR.

While causal mechanisms could not be constructed in relation to these final solution paths, it is still worth discussing some of the results to come out of the process tracing efforts. For example, it was found that T&C employment loss in the Kyrgyz Republic was largely offset by T&C employment growth within the informal sector [82]. Moreover while the Kyrgyz Republic is characterized in the fsQCA as having protective labour market policies, any workers losing their formal employment were unlikely to have been able to access related social provision [83]. One reason for this is that workers were likely to be employed in smaller establishments and, as in Italy, labour regulations exclude from their provisions firms with fewer than 15 employees [63]. In relation to China and Thailand, results were very similar to those discussed in relation to Bangladesh, employment growth was found to have occurred both in the context of poor working conditions and weak labour market and social provisions [84–86]. In Thailand however, growth in T&C employment was seen to occur mainly in informal and migrant labour [87]. Finally, both Sri Lanka and Indonesia were found to have some form of social protection aimed at T&C workers, despite overall poor working conditions [88, 89]. In both countries, this protection comes from employment contribution schemes whereby workers are able to withdraw benefits under various circumstances related for instance to retirement, employment loss and medical reasons. However, the degree to which these regulations are adhered to is suspect [90].

## Discussion

Aligned with previous comparative welfare state studies [10], the results of this work seem to indicate the health importance of protective social policies. Potential causal mechanisms emerged for two solution paths and suggest that a worsening of AFM after the MFA phase-out is related to T&C workers’ inability to access social protection. This is found to be the case in the context of both T&C employment growth (in less developed countries) and loss (in highly developed countries).

Across the typical cases in these solution paths, T&C workers were found to have little access to protective social policies, regardless of how countries’ were characterized in the fsQCA. This indicates that the fsQCA conditions used to measure social protection were inappropriate for the T&C industry, although they were chosen on the basis of externally available data. Relatedly, since the potential mechanisms arising out of this work focus on conditions which differ from those of the fsQCA, claims about their sufficiency cannot be made. While this represents a potential limitation of this work, it is also a major finding that social protection may be inaccessible to the type of workers who are most vulnerable to processes of liberalization, even when a country can be characterized as having broadly protective policies. For example, despite characterizations of relatively protective labour market policies in Italy and the Kyrgyz Republic, T&C workers in these countries who were found to be the most likely to lose their employment after the MFA phase-out, were employed in precisely the type of firms excluded from these provisions.

Evidence collected in relation to the first solution path also points to the health importance of protective social policies despite the fact that a causal mechanism could not be constructed. Here we encounter evidence from Brazil where reductions in poverty, inequality and child mortality have been associated with an expansive conditional cash transfer programme.

No condition was found to be necessary for any of the health outcomes. This is unsurprising since necessary causes are understood to be a rare empirical event [29]. In terms of sufficiency, the MFA phase-out wasn’t found to be related to countries’ changing IMR or improving AFM. However, this does not indicate that the phase-out did not impact these outcomes. Indeed, limited data on T&C workers was a primary reason for the difficulty in tying the MFA phase-out to changing IMR. Moreover, this work likely underestimates the health impact of the MFA phase-out since factors outside of labour markets are not considered (e.g., changes in industrial pollution).

In the case of improving AFM, the fsQCA solution paths incorporated only one of the 10 cases exhibiting this outcome (i.e., Brazil). Improvements in social circumstances may be involved in the other nine countries exhibiting improving AFM, but additional case study work is needed to further investigate this possibility.

Aside from aiding in the construction of two potential causal mechanisms, combining fsQCA with in-depth country studies also aided in the development of theory, both in relation to the specific health impact of the MFA phase-out and to the relationship between trade liberalization and health more broadly. In the context of the MFA phase-out, trade liberalization was found to shift both employment and working conditions. Changes in employment were found to extend beyond strict gains or losses and encompass a general move to more precarious conditions. For example, loss of formal T&C employment in the Kyrgyz Republic was found to correspond with employment growth in the informal sector. Employment growth in Thailand’s T&C sector was found to mask a greater reliance on migrant and informal employment. Furthermore, in both countries experiencing employment growth (e.g., Bangladesh) and loss (e.g., Italy), evidence was found for a worsening of T&C working conditions after the MFA phase-out.

Social policies were found to interact with these considerations after the MFA phase-out, and in response to the conditions of the sector in general, in many health important ways. Factors such as the size of T&C firms and employees’ labour contracts were found to determine workers’ access to social protection. These conditions often in turn, relate back to countries’ labour regulations. In other words, social policies were found to both moderate pathways to health in the context of labour markets and influence the type of health-related pathways resulting from trade liberalization. They were found to moderate pathways to health by influencing the type of social protection available to workers. They were found to influence the type of health-related pathways resulting from trade liberalization by shaping factors such as the type of employment contracts through which firms hire workers. Interestingly, social protection for T&C workers in some less developed countries seemed to be greater than in other more developed countries.

## Conclusion

This study is the first to empirically link trade liberalization to employment as an important SDH. It also offers some of the first empirical insights on how trade liberalization interacts with social policies to influence health and in doing so, demonstrates the merits of a configurational methodological approach. While still preliminary, findings are aligned with other work which points to the health importance of social protection policies. A major finding of this work is that social protection may be inaccessible to the type of workers who are vulnerable to processes of liberalization, even when this protection is available to a country’s population at large, and that workers can be particularly vulnerable to processes of liberalization due to the structure of their country’s social policies.

## Additional file

Additional file 1: Data and corresponding fuzzy-set scores for each of the outcomes and causal conditions. (DOCX 33 kb)

## Abbreviations

AFM: adult female mortality rates
EMCONET: Employment Conditions Knowledge Network
fsQCA: fuzzy-set qualitative comparative analysis
HDI: Human Development Index
ILO: International Labor Organization
IMR: infant mortality rates
MFA: Multi-Fibre Arrangement
QCA: qualitative comparative analysis
SDH: social determinants of health
T&C: textile and clothing
UNIDO: United Nations Industrial Development Organization
