# Supplementary material for: Trade liberalization, social policies and health: an empirical case study
Source: Global Health. 2015 Oct 12;11:42. doi: 10.1186/s12992-015-0126-8 (PMC4601122; doi:10.1186/s12992-015-0126-8)
Supplement: Additional file 1: — Data and corresponding fuzzy-set scores for each of the outcomes and causal conditions. (DOCX 33 kb) [file 12992_2015_126_MOESM1_ESM.docx]

**Additional file 1**

Fuzzy-set membership scores for the outcome sets of Health Improving and Health Worsening adult female and infant mortality rates

| Country | Difference in adult female mortality rate reductions between pre (2000-2004) and post (2005-2009) MFA periods | Fuzzy Score in Health Improving Set  (AFM) | Fuzzy Score in Health Worsening Set  (AFM) | Difference in infant mortality rate reductions between pre and post MFA periods | Fuzzy Score in Health Improving Set  (IMR) | Fuzzy Score in Health Worsening Set  (IMR) |
| --- | --- | --- | --- | --- | --- | --- |
| Azerbaijan | NA | NA | NA | 3.71 | 0.94 | 0.06 |
| Bangladesh | -4.28 | 0.01 | 0.99 | -2.46 | 0.14 | 0.86 |
| Brazil | 2.74 | 0.94 | 0.06 | -0.52 | 0.4 | 0.6 |
| Bulgaria | -2.33 | 0.09 | 0.91 | -6.38 | 0.01 | 0.99 |
| China | -1.37 | 0.2 | 0.8 | -7.32 | 0 | 1 |
| Colombia | -1.30 | 0.21 | 0.79 | 3.92 | 0.95 | 0.05 |
| Croatia | -8.67 | 0 | 1 | 5.80 | 0.99 | 0.01 |
| Ecuador | 1.12 | 0.75 | 0.25 | 1.37 | 0.74 | 0.26 |
| Egypt | NA | NA | NA | 0.53 | 0.6 | 0.4 |
| Estonia | -3.18 | 0.04 | 0.96 | NA | NA | NA |
| Greece | 3.50 | 0.97 | 0.03 | 7.22 | 1 | 0 |
| Hungary | -3.91 | 0.02 | 0.98 | NA | NA | NA |
| India | -2.87 | 0.05 | 0.95 | -0.40 | 0.43 | 0.57 |
| Indonesia | NA | NA | NA | 0.31 | 0.56 | 0.44 |
| Italy | -5.91 | 0 | 1 | -21.84 | 0 | 1 |
| Korea | -2.56 | 0.07 | 0.93 | 13.58 | 1 | 0 |
| Kyrgyz Republic | -4.80 | 0.01 | 0.99 | 2.03 | 0.82 | 0.18 |
| Latvia | -5.26 | 0.01 | 0.99 | -14.99 | 0 | 1 |
| Lithuania | -7.02 | 0 | 1 | 0.31 | 0.56 | 0.44 |
| Mauritius | 0.024 | 0.51 | 0.49 | -15.78 | 0 | 1 |
| Mexico | -0.250 | 0.44 | 0.56 | 0.02 | 0.5 | 0.5 |
| Morocco | NA | NA | NA | 0.72 | 0.63 | 0.37 |
| Peru | 9.24 | 1 | 0 | -2.47 | 0.14 | 0.86 |
| Philippines | 4.27 | 0.99 | 0.01 | 2.65 | 0.88 | 0.12 |
| Poland | -8.64 | 0 | 1 | -8.89 | 0 | 1 |
| Portugal | 0.63 | 0.65 | 0.35 | -19.89 | 0 | 1 |
| Romania | 0.32 | 0.58 | 0.42 | NA | NA | NA |
| Slovak Republic | -1.93 | 0.13 | 0.87 | 8.31 | 1 | 0 |
| South Africa | 34.62 | 1 | 0 | 46.75 | 1 | 0 |
| Sri Lanka | NA | NA | NA | -10.29 | 0 | 1 |
| Thailand | 3.83 | 0.98 | 0.02 | 0.13 | 0.53 | 0.47 |
| Turkey | -4.05 | 0.02 | 0.98 | 2.12 | 0.83 | 0.17 |

Fuzzy-set membership scores in the set of Highly Developed Countries

| Country | HDI Index Score (2004) | Fuzzy Score |
| --- | --- | --- |
| Azerbaijan | 0.736 | 0.35 |
| Bangladesh | 0.53 | 0.06 |
| Brazil | 0.792 | 0.48 |
| Bulgaria | 0.816 | 0.62 |
| China | 0.768 | 0.42 |
| Colombia | 0.79 | 0.48 |
| Croatia | 0.846 | 0.8 |
| Ecuador | 0.765 | 0.41 |
| Egypt | 0.702 | 0.27 |
| Estonia | 0.858 | 0.85 |
| Greece | 0.921 | 0.97 |
| Hungary | 0.869 | 0.89 |
| India | 0.611 | 0.13 |
| Indonesia | 0.711 | 0.29 |
| Italy | 0.94 | 0.99 |
| Korea | 0.912 | 0.97 |
| Kyrgyz Republic | 0.705 | 0.28 |
| Latvia | 0.845 | 0.79 |
| Lithuania | 0.857 | 0.85 |
| Mauritius | 0.8 | 0.5 |
| Mexico | 0.821 | 0.65 |
| Morocco | 0.64 | 0.17 |
| Peru | 0.767 | 0.42 |
| Philippines | 0.763 | 0.41 |
| Poland | 0.862 | 0.87 |
| Portugal | 0.904 | 0.96 |
| Romania | 0.805 | 0.54 |
| Slovak Republic | 0.856 | 0.84 |
| South Africa | 0.653 | 0.19 |
| Sri Lanka | 0.755 | 0.39 |
| Thailand | 0.784 | 0.46 |
| Turkey | 0.757 | 0.39 |

Fuzzy-set membership scores in the set of Protective Labour Market Policies

| **Country** | **Freedom of association** | | **Forced labour** | | **Discrimination** | | **Child labour** | | **Fuzzy Score** |
| --- | --- | --- | --- | --- | --- | --- | --- | --- | --- |
|  | C087 | C098 | C029 | C105 | C100 | C111 | C138 | C182 |  |
| Azerbaijan | 1992 | 1992 | 1992 | 2000 | 1992 | 1992 | 1992 | 2004 | 1 |
| Bangladesh | 1972 | 1972 | 1972 | 1972 | 1998 | 1972 |  | 2001 | 0.6 |
| Brazil |  | 1952 | 1957 | 1965 | 1957 | 1965 | 2001 | 2000 | 0.6 |
| Bulgaria | 1959 | 1959 | 1932 | 1999 | 1955 | 1960 | 1980 | 2000 | 1 |
| China |  |  |  |  | 1990 | 2006 | 1999 | 2002 | 0.2 |
| Colombia | 1976 | 1976 | 1969 | 1963 | 1963 | 1969 | 2001 | 2005 | 0.8 |
| Croatia | 1991 | 1991 | 1991 | 1997 | 1991 | 1991 | 1991 | 2001 | 1 |
| Ecuador | 1967 | 1959 | 1954 | 1962 | 1957 | 1962 | 2000 | 2000 | 1 |
| Egypt | 1957 | 1954 | 1955 | 1958 | 1960 | 1960 | 1999 | 2002 | 1 |
| Estonia | 1994 | 1994 | 1996 | 1996 | 1996 | 2005 | 2007 | 2001 | 1 |
| Greece | 1962 | 1962 | 1952 | 1962 | 1975 | 1984 | 1986 | 2001 | 1 |
| Hungary | 1957 | 1957 | 1956 | 1994 | 1956 | 1961 | 1998 | 2000 | 1 |
| India |  |  | 1954 | 2000 | 1958 | 1960 |  |  | 0.2 |
| Indonesia | 1998 | 1957 | 1950 | 1999 | 1958 | 1999 | 1999 | 2000 | 1 |
| Italy | 1958 | 1958 | 1934 | 1968 | 1956 | 1963 | 1981 | 2000 | 1 |
| Korea |  |  |  |  | 1997 | 1998 | 1999 | 2001 | 0.2 |
| Kyrgyz Republic | 1992 | 1992 | 1992 | 1999 | 1992 | 1992 | 1992 | 2004 | 1 |
| Latvia | 1992 | 1992 | 2006 | 1992 | 1992 | 1992 | 2006 | 2006 | 0.8 |
| Lithuania | 1994 | 1994 | 1994 | 1994 | 1994 | 1994 | 1998 | 2003 | 1 |
| Mauritius | 2005 | 1969 | 1969 | 1969 | 2002 | 2002 | 1990 | 2000 | 0.8 |
| Mexico | 1950 |  | 1934 | 1959 | 1952 | 1961 |  | 2000 | 0.4 |
| Morocco |  | 1957 | 1957 | 1966 | 1979 | 1963 | 2000 | 2001 | 0.6 |
| Peru | 1960 | 1964 | 1960 | 1960 | 1960 | 1970 | 2002 | 2002 | 1 |
| Philippines | 1953 | 1953 | 2005 | 1960 | 1953 | 1960 | 1998 | 2000 | 0.8 |
| Poland | 1957 | 1957 | 1958 | 1958 | 1954 | 1961 | 1978 | 2002 | 1 |
| Portugal | 1977 | 1964 | 1956 | 1959 | 1967 | 1959 | 1998 | 2000 | 1 |
| Romania | 1957 | 1958 | 1957 | 1998 | 1957 | 1973 | 1975 | 2000 | 1 |
| Slovakia | 1993 | 1993 | 1993 | 1997 | 1993 | 1993 | 1997 | 1999 | 1 |
| South Africa | 1996 | 1996 | 1997 | 1997 | 2000 | 1997 | 2000 | 2000 | 1 |
| Sri Lanka | 1995 | 1972 | 1950 | 2003 | 1993 | 1998 | 2000 | 2001 | 1 |
| Thailand |  |  | 1969 | 1969 | 1999 |  | 2004 | 2001 | 0.2 |
| Turkey | 1993 | 1952 | 1998 | 1961 | 1967 | 1967 | 1998 | 2001 | 1 |

Fuzzy-set membership scores in the set of Protective Welfare State Policies

| Country | ISI (2004) | Category | Fuzzy Score |
| --- | --- | --- | --- |
| Azerbaijan | 0.424 | Much to be done | 0 |
| Bangladesh | 0.365 | Much to be done | 0 |
| Brazil | 0.586 | Conventional | 0.67 |
| Bulgaria | 0.658 | Conventional | 0.67 |
| China | 0.428 | Much to be done | 0 |
| Colombia | 0.335 | Much to be done | 0 |
| Croatia | 0.679 | Conventional | 0.67 |
| Ecuador | 0.464 | Conventional | 0.67 |
| Egypt | 0.505 | Much to be done | 0 |
| Estonia | 0.627 | Pragmatist | 0.33 |
| Greece | 0.594 | Pragmatist | 0.33 |
| Hungary | 0.672 | Pragmatist | 0.33 |
| India | 0.288 | Much to be done | 0 |
| Indonesia | 0.328 | Much to be done | 0 |
| Italy | 0.681 | Pragmatist | 0.33 |
| Korea | 0.666 | Pragmatist | 0.33 |
| Kyrgyz Republic | 0.371 | Much to be done | 0 |
| Latvia | 0.694 | Pacesetter | 1 |
| Lithuania | 0.622 | Pragmatist | 0.33 |
| Mauritius | 0.654 | Conventional | 0.67 |
| Mexico | 0.555 | Conventional | 0.67 |
| Morocco | 0.331 | Much to be done | 0 |
| Peru | 0.356 | Much to be done | 0 |
| Philippines | 0.432 | Conventional | 0.67 |
| Poland | 0.692 | Pacesetter | 1 |
| Portugal | 0.738 | Pacesetter | 1 |
| Romania | 0.514 | Conventional | 0.67 |
| Slovakia | 0.626 | Pacesetter | 1 |
| South Africa | 0.487 | Conventional | 0.67 |
| Sri Lanka | 0.502 | Conventional | 0.67 |
| Thailand | 0.408 | Much to be done | 0 |
| Turkey | 0.567 | Conventional | 0.67 |

Fuzzy-set membership scores in the sets of Employment Growth and Employment Loss

| Country | Years | Change in Employment (percentage) | Fuzzy-set Employment Growth | Fuzzy-set Employment Loss |
| --- | --- | --- | --- | --- |
| Azerbaijan | 2004-2008 | -19.78 | 0 | 0.99 |
| Bangladesh | 2004-2008* | 40.00 | 1 | 0 |
| Brazil | 2004-2007 | 11.86 | 0.89 | 0 |
| Bulgaria | 2004-2008 | -17.88 | 0 | 0.98 |
| China | 2004-2008 | 18.03 | 0.98 | 0 |
| Colombia | 2004-2005 | -4.07 | 0 | 0.36 |
| Croatia | 2004-2008 | -18.06 | 0 | 0.98 |
| Ecuador | 2004-2008 | 3.04 | 0.24 | 0.01 |
| Egypt | 2004-2006 | -1.53 | 0.02 | 0.11 |
| Estonia | 2004-2008 | -34.05 | 0 | 1 |
| Greece | 2004-2008 | -3.49 | 0.01 | 0.29 |
| Hungary | 2004-2008 | -41.56 | 0 | 1 |
| India | 2004-2008 | 21.31 | 0.99 | 0 |
| Indonesia | 2004-2008 | 8.62 | 0.75 | 0 |
| Italy | 2004-2008 | -13.29 | 0 | 0.92 |
| Korea | 2004-2006 | -12.17 | 0 | 0.9 |
| Kyrgyz Republic | 2004-2008 | -27.22 | 0 | 1 |
| Latvia | 2004-2008 | -32.26 | 0 | 1 |
| Lithuania | 2004-2008 | -39.01 | 0 | 1 |
| Mauritius | 2004-2008 | -14.82 | 0 | 0.95 |
| Mexico | 2004-2008* | -35.00 | 0 | 1 |
| Morocco | 2004-2008 | -8.37 | 0 | 0.73 |
| Peru | 2004-2008 | -16.20 | 0 | 0.97 |
| Philippines | 2003-2006 | -6.75 | 0 | 0.63 |
| Poland | 2004-2008 | -8.42 | 0 | 0.74 |
| Portugal | 2004-2008 | -12.05 | 0 | 0.89 |
| Romania | 2004-2008 | -39.96 | 0 | 1 |
| Slovakia | 2004-2008 | -21.24 | 0 | 0.99 |
| South Africa | 2004-2008 | -32.16 | 0 | 1 |
| Sri Lanka | 2001-2008 | 80.51 | 1 | 0 |
| Thailand | 2002-2006 | 8.06 | 0.71 | 0 |
| Turkey | 2004-2006 | -0.29 | 0.04 | 0.06 |

*Since data was not available on Bangladesh and Mexico in the UNIDO database, and because these countries are oft cited as extremely reliant on the textile and clothing sector, data was taken from the World Bank (Lopez-Acevedo & Robertson, 2012).
